# Efficacy and tolerability of lithium in treating acute mania in youth with bipolar disorder: protocol for a systematic review

**DOI:** 10.1186/s40345-017-0092-6

**Published:** 2017-06-13

**Authors:** A. Duffy, S. Patten, S. Goodday, A. Weir, N. Heffer, A. Cipriani

**Affiliations:** 1 Mood Disorders Centre of Ottawa, University of Ottawa Health Services, 100 Marie Curie Private, Suite 300, Ottawa, ON K1N 6N5 Canada; 2 Department of Psychiatry, Hotel Dieu Hospital, Queen’s University, 166 Brock Street, Kingston, ON K7L 5G2 Canada; 3 Department of Psychiatry, University of Calgary, Calgary, AB Canada; 4 Department of Epidemiology, University of Toronto, Toronto, ON Canada; 5 Department of Epidemiology, University of Ottawa, Ottawa, ON Canada; 6 Oxford University, Oxford, UK; 7 Department of Psychiatry, Oxford University, Oxford, UK; 8 Oxford Health NHS Foundation Trust, Warneford Hospital, Oxford, OX3 7JX UK

**Keywords:** Lithium, Mania, Child and adolescent, Systematic review, Bipolar disorder, Acute treatment

## Abstract

**Background:**

Epidemiological, clinical, and high-risk studies have provided evidence that the peak period for onset of diagnosable episodes of mania and hypomania starts in mid-to-late adolescence. Moreover, clinically significant manic symptoms may occur even earlier, especially in children at familial risk. Lithium is the gold standard treatment for acute mania in adults, yet to our knowledge, there is no published systematic review assessing lithium treatment of mania in children or adolescents. This is a major gap in knowledge needed to inform clinical practice.

**Aim:**

As a working group within the ISBD Task Force on Lithium Treatment (http://www.isbd.org/active-task-forces), our aim is to complete a systematic review of the efficacy, tolerability, and acceptability of lithium compared with placebo and other active drugs in treating mania in children and adolescents diagnosed with bipolar disorder.

**Methods:**

We will include double- or single-blind randomized controlled trials in patients aged less than 18 years. No restrictions will be made by study publication date or language. Several electronic databases will be searched along with secondary sources such as bibliographies and trial registry websites for published and unpublished studies. Response rates to lithium compared with placebo or other active drugs will be the primary efficacy outcome. Primary tolerability and acceptability outcomes will be rates of serious adverse events and dropouts, respectively. Secondary outcomes will include rates of remission, severity of manic symptoms at different time points, and incidence of specific adverse events.

**Discussion:**

Findings from this systematic review are critically needed to inform clinical practice. We should not generalize findings from adult studies, as children and adolescents are undergoing accelerated physiological and brain development. Therefore, efficacy, tolerability, and acceptability of lithium treatment of acute mania in children compared to adults may be very different. This systematic review has been registered in PROSPERO (CRD42017055675).

## Background

Bipolar disorder (BD) describes a group of heterogeneous mood disorders (Angst et al. 2004). More than 2% of the world’s population is affected with the most severe forms identified by a manic, mixed or hypomanic episode; while an estimated 5% of the population is affected with milder spectrum conditions (McDonald et al. 2015; Merikangas et al. 2007). In addition, relatively high rates of manic symptoms are reported in child and adolescent non-clinical populations, which can represent normative variants rather than precursors to BD unless combined with other risk factors such as family history (Tijssen et al. 2010a, b). Bipolar disorder runs in families with an estimated heritability of up to 80% (Bienvenu et al. 2011; Smoller and Finn 2003). High-risk, clinical, and population studies estimate that the peak period for onset of BD is adolescence and early adulthood (Duffy et al. 2014; Mesman et al. 2013; Leboyer et al. 2005; Angst et al. 2005a, b). Yet, it is estimated to take over a decade to accurately diagnose BD and this delay is associated with devastating consequences including school drop-out, economic, occupational, and interpersonal problems, inappropriate treatment, substance abuse and chronicity (Ghaemi et al. 2002; Judd et al. 2005). Given that the onset occurs during a critical developmental period, taken together with the lag in diagnosis and severity of acute episodes early in the course, it is not surprising that BD is among the leading causes of years lived with disability worldwide (Whiteford et al. 2013). Further, there is a significant reduction in life expectancy evident already early in the illness course in adolescent patients attributable to medical illness, accidents, and suicide (Baethge and Cassidy 2013; Kessing et al. 2015a; b).

Lithium is the first line or gold standard treatment for acute mania and prophylaxis of recurrent BD episodes (both manic and depressive) in adults (Yatham et al. 2013; Grof and Muller-Oerlinghausen 2009). Moreover, substantial evidence supports that lithium has a specific anti-suicidal effect with the potential to normalize the morbidity rate in BD patients (Muller-Oerlinghausen et al. 1992; Cipriani et al. 2013; Baldessarini and Tondo 2008). Despite the widespread use of lithium in treating BD for over 50 years, we still know little about the specific therapeutic mechanisms of action (Malhi and Outhred 2016). Importantly here, lithium started earlier in the course of BD may have a higher likelihood of treatment response (Kessing et al. 2014) and preliminary findings suggest that lithium may have neuroprotective effects (Malhi and Outhred 2016; Hajek et al. 2013; Pfennig et al. 2014). Yet, the effectiveness, tolerability, and acceptability of lithium treatment in children and adolescents diagnosed with BD is even less well understood and understudied. As a result, most guidelines focus on adult patients with established illness (Yatham et al. 2013; Fountoulakis et al. 2016). Therefore, as part of the ISBD Task Force on Lithium Treatment (http://www.isbd.org/active-task-forces), our working group is embarking on a systematic review of studies to inform efficacy, tolerability, and acceptability of lithium treatment for acute mania in children and adolescents diagnosed with BD.

## Methods

### Criteria for considering studies

We will include double- or single-blind randomized controlled trials where lithium is used in the treatment of acute manic episodes in comparison with other active drugs or placebo. In view of the important role for randomization as a methodological protection against selection bias and confounding, we will exclude all quasi-randomized studies. Studies that report randomization but do not report a procedure for random assignment will be included, as adequacy of randomization will be quantified in our risk of bias assessment. For trials that have a cross-over design, we will only consider results from the first period prior to cross-over.

### Participants

We will consider all studies including males and females less than 18 years of age with a primary diagnosis of BD and experiencing a manic episode, according to standard diagnostic criteria such as DSM or equivalent. Studies that defined mania only as scoring above a certain cut-off on a screening questionnaire will be excluded, as will studies that defined a manic equivalent as part of a primary mood dysregulation disorder. We will not apply any restrictions by treatment setting. We will not consider concurrent secondary diagnosis of another psychiatric disorder an exclusion criterion. However, we will exclude studies recruiting participants with a serious concomitant medical illness, neurological disorder, diagnosed intellectual disability, or brain injury.

### Interventions

#### Experimental

- Lithium any dose within the therapeutic range (between 0.4 and 1.2 mmol/l) and any method of administration (i.e. tablet or syrup).

#### Comparator

- Placebo.
- Any other active drugs tested for acute mania (including atypical and typical antipsychotics and anticonvulsants).

All interventions could be monotherapy or combined with other treatments. We will include trials that allow for rescue medications (as required, short-term, infrequent use aimed at emergent symptom relief only) as long as these medications were equally applied among the randomized arms.

### Outcome measures

We will include studies that meet the above inclusion criteria regardless of whether they reported on the outcomes under study.

#### Primary outcomes

- *Efficacy*: Difference in response (as defined by a decrease in score on any validated mania rating scale of ≥50% from baseline) between lithium and comparatively treated patients (placebo or other anti-manic agent) at designated time points
- *Tolerability*: Difference in serious adverse events (e.g. death, renal failure, diabetes insipidus, clinically significant ECG changes, toxic rash) between lithium and comparatively treated patients
- *Acceptability*: Differences in discontinuation rates for any reason between lithium and comparatively treated patients

#### Secondary outcomes

- *Efficacy*: Difference in remission (YMRS (Young et al. 1978) score of ≤12 or equivalent) between lithium and comparatively treated patients at designated time points
- *Efficacy*: Difference in mean endpoint scores and change in scores of manic symptoms (as measured by the YMRS or equivalent)
- *Tolerability*: Differences in specific side effects including but not limited to cognitive impairment, diarrhoea, gastric irritation, nausea, haematological abnormalities, hypothyroidism, hyperparathyroidism, polyuria, non-toxic rash, somnolence, lethargy, thirst, tremor, weight gain

To avoid missing any rare or unexpected side effects, in the data-extraction phase, we will collect information on all side effects reported in the included studies and discuss ways to summarize them post hoc.

Outcomes will be recorded at the following time points, if reported by individual studies:

- At 4 days (if not available less than 1 week)
- At 1 week (or between 1 and 2 weeks)
- At 3 weeks (or more than 2 and up to 4 weeks)
- At 6 weeks (or more than 4 and up to 8 weeks)
- At 12 weeks (or more than 8 and up to 16 weeks)

### Search methods for identification of studies

We will search EMBASE, MEDLINE, PsycINFO, CINAHL, and the Cochrane Central Register of Controlled Trials (CENTRAL), the trial databases of regulatory agencies and the websites of pharmacological industries for published, unpublished and ongoing randomized controlled trials. No language or study publication date restrictions will be applied. See PROSPERO (CRD42017055675) for full details about the search strategy, including the text words and keywords that will be used and the list of websites.

### Data extraction and analysis

Two authors (AW and NH) will independently screen titles and abstracts to identify potentially relevant studies retrieved by the search strategy. The full text of the screened studies will be reviewed for inclusion. Agreement rates on the initial assessments will be reported using the kappa coefficient. All reasons for excluding the ineligible studies will be recorded. Any disagreement will be resolved through discussion or, if required, by consulting other authors of the review team (AD, SP, AC). The frequency and nature of all such disagreements will be recorded in a study log. We will identify and remove duplicate records and collate multiple reports that relate to the same study so that each study, rather than each report, is the unit of interest in the review. We will record the selection process in sufficient detail to complete a PRISMA (Moher et al. 2009) flow diagram and a characteristics of excluded studies table.

### Assessment of risk of bias in included studies

Pairs of review authors (AD, AW, SP, SG) will independently assess the risks of bias for each study using the criteria outlined in the Cochrane Handbook for Systematic Reviews of Interventions (Higgins et al. 2011; Higgins and Green 2011). Any disagreements will be resolved by discussion with another member of the review team (AC) and the results of these discussions will be logged. We will assess the risk of bias according to the following domains: random sequence generation, allocation concealment, blinding of participants and personnel, blinding of outcome assessment, incomplete outcome data, and selective outcome reporting. We will judge each potential source of bias as high, low, or unclear. Intra-class correlation coefficients will be used to quantify the risk of bias assignments by different reviewers.

### Measures of treatment effect

#### Continuous data

We will calculate the mean difference (MD) or standardized mean difference (SMD) along with corresponding 95% confidence intervals (CI) for continuous outcomes. We will use the MD where the same scale is used to measure an outcome. We will employ the SMD where different scales were used to measure the same underlying construct.

#### Dichotomous data

We will calculate the relative risk (RR) with corresponding 95% CI for dichotomous outcomes. We will calculate response rates out of the total number of randomized participants. For statistically significant results, we will calculate the number needed to treat for an additional beneficial outcome (NNTB) and the number needed to treat for an additional harmful outcome (NNTH).

For both continuous and dichotomous data, we will only conduct a meta-analysis if pooling is appropriate; that is, if the treatments, participants, and the underlying clinical question are homogenous enough. We will narratively describe skewed data reported as medians and interquartile ranges. If meta-analysis is pursued, log transformation of relative risks will be used to enhance normality of the estimates. Standard errors will be estimated from the reported confidence intervals.

### Missing data

We will contact the original study authors for missing data when these are inadequately described in the study publications.

#### Missing dichotomous data

We will calculate responders to treatment and remitters on a strict intention-to-treat (ITT) basis and we will include dropouts in this analysis. Where participants were excluded from a trial before the endpoint, we will assume that they experienced a negative outcome by the end of the trial (failure to respond to treatment). We will examine the validity of this decision in sensitivity analyses.

#### Missing continuous data

When there are missing data and the method of ‘last observation carried forward’ (LOCF) is used to perform an ITT analysis, we will use the LOCF data. When only the standard error or *t* statistics or *p* values are reported, we will calculate standard deviations (SD) according to Altman and Bland (1996). Where SDs are not reported, we will contact the authors for these data but, in the absence of data from the authors, we will borrow SD from other studies in the review (Furukawa et al. 2006). We will examine the validity of this imputation by sensitivity analyses.

### Assessment of heterogeneity

We will first investigate heterogeneity between studies by visual inspection of all forest plots. If the 95% CI’s of the RR’s for each study in the pooled analysis does not include means of other included studies, we will investigate potential sources of heterogeneity. We will also calculate the *I* ^2^ statistic and will interpret the level of heterogeneity according to the criteria outlined by the Cochrane Handbook for Systematic Reviews of Interventions (2011). We will consider if the importance of the observed value of *I* ^2^ depends on (i) the magnitude and direction of effects and (ii) the strength of evidence for heterogeneity.

### Assessment of reporting biases

We will assess publication bias and small-study effects using a funnel plot. We plan to use the test for funnel plot asymmetry only when at least 10 studies are included in the meta-analysis. In the event of using a funnel plot, we will interpret results cautiously using visual inspection. If we identify evidence of small-study effects, we will investigate possible reasons for funnel plot asymmetry including publication bias (Egger et al. 1997).

### Data synthesis

We will calculate the pooled RR with corresponding 95% CIs for dichotomous outcomes. We will calculate the pooled MD or SMD as appropriate with corresponding 95% CIs for continuous outcomes. Statistical significance will be defined as a *p* value of less than 0.05 and a 95% CI that does not cross the line of no effect. In forest plots with two or more studies, we will use a random-effects model for both dichotomous and continuous variables. We will adopt the random effects model under these circumstances because it has the highest generalizability for empirical examination of summary effect measures in meta-analyses (Furukawa et al. 2002).

### Subgroup analysis

As multiple analyses can lead to false-positive and false-negative conclusions, subgroup analyses should be performed and interpreted with caution. We will conduct subgroup analyses for primary outcomes comparing children (up to 12 years) to adolescents (between 13 and 18 years).

### Sensitivity analysis

We will conduct the following sensitivity analyses for primary outcomes:

- Excluding trials with unclear allocation concealment or unclear double-blinding
- Excluding studies that recruited participants with rapid-cycling
- Excluding trials with a dropout rate greater than 20%
- Excluding trials for which the SD had to be borrowed from other trials
- Excluding trials where lithium was the comparator drug
- Excluding trials with high-risk of bias from any source
- Excluding cross-over trials where only first period data are available

We will explore sensitivity analyses for missing data by applying worst and best case scenarios (that is, missing data are assumed either as responder or non-responder in the corresponding sensitivity analysis). Finally, we will explore potential additional sensitivity analyses and will report these post hoc.

### Summary of findings table

We will construct a ‘Summary of findings’ table for each comparison and use GRADE proGDT software and the principles of the GRADE approach (Atkins et al. 2004) to assess the quality of a body of evidence based on the extent to which there can be confidence that the obtained effect estimate reflects the true underlying effect.

## Discussion

There is a major evidence gap needed to inform clinicians faced with treating seriously ill youth diagnosed with BD. Over the past decade, there have been a number of individual acute mania treatment studies published that should be systematically reviewed and if possible findings combined into a meta-analysis. While more, well-designed, treatment trials are needed in larger numbers of BD youth during various phases of the illness, this systematic review represents an important next step; summarizing the available data to provide evidence about the efficacy, tolerability, and acceptability of lithium treatment of acute mania in youth with a primary diagnosis of bipolar disorder.
